# The Effect of Mycophenolate Mofetil on Disease Development in the *gld.apoE* ^−/−^ Mouse Model of Accelerated Atherosclerosis and Systemic Lupus Erythematosus

**DOI:** 10.1371/journal.pone.0061042

**Published:** 2013-04-08

**Authors:** Christophe Richez, Rocco J. Richards, Pierre Duffau, Zachary Weitzner, Christopher D. Andry, Ian R. Rifkin, Tamar Aprahamian

**Affiliations:** 1 Service de Rhumatologie, Hôpital Pellegrin, CHU de Bordeaux, Bordeaux, France; 2 UMR-CNRS 5164, Université Victor Segalen Bordeaux 2, Bordeaux, France; 3 Department of Medicine-Renal Section, Boston University School of Medicine, Boston, Massachusetts, United States of America; 4 Service de Médecine Interne, Hôpital Saint André, CHU de Bordeaux, Bordeaux, France; 5 Department of Medicine-Pathology, Boston University School of Medicine, Boston, Massachusetts, United States of America; Baylor College of Medicine, United States of America

## Abstract

Systemic lupus erythematosus (SLE) is a systemic autoimmune disease that is characterized by autoantibody production and inflammatory disease involving multiple organs. Premature atherosclerosis is a common complication of SLE and results in substantial morbidity and mortality from cardiovascular disease (CVD). The reasons for the premature atherosclerosis in SLE are incompletely understood, although chronic inflammation is thought to play an important role. There is currently no known preventative treatment of premature atherosclerosis in SLE. Mycophenolate mofetil (MMF) is an immunosuppressive agent that is commonly used for treatment of patients with SLE. In order to study the impact of this drug on murine lupus disease including premature atherosclerosis development, we treated *gld.apoE^−/−^* mice, a model of SLE and accelerated atherosclerosis, with MMF. We maintained seven-week old *gld.apoE^−/−^* mice on a high cholesterol Western diet with or without MMF. After 12 weeks on diet, mice receiving MMF showed decreased atherosclerotic lesion area compared to the control group. MMF treatment also improved the lupus phenotype, indicated by a significant decrease circulating autoantibody levels and ameliorating lupus nephritis associated with this model. This data suggests that the effects of MMF on the immune system may not only be beneficial for lupus, but also for inflammation driving lupus-associated atherosclerosis.

## Introduction

Systemic lupus erythematosus (SLE) is a complex systemic autoimmune disease involving multiple organs that is characterized by autoantibody production and chronic inflammation [1]. Over time, management of SLE patients has improved and life expectancy of these patients has increased to reach a 10-year survival rate about 70% [2]. However, several studies have revealed that atherosclerosis-attributed vascular events are significantly more frequent in these surviving lupus patients, compared to age-related individuals without SLE [3], [4].

Atherosclerosis is characterized by a chronic inflammatory state where immune cell activity is linked to plaque formation and remodeling [5]. A plaque is formed within the lumen of medium- and large-sized arteries due to physiological imbalances caused by chronic inflammation; the plaque is described as a progressive accumulation of lipid, inflammatory cells, smooth muscle cells, and connective tissue within the intima of arteries [6]. It has become widely accepted that atherosclerosis is an inflammatory disease, and that the immune system plays a pivotal role in disease development. Therefore, it is reasonable to suggest that the chronic inflammatory condition encountered in SLE and the activation of immune cells may predispose patients to an increased risk of premature atherosclerosis leading to cardiovascular disease (CVD). For these reasons, immunomodulatory therapy might be of benefit in ameliorating atherosclerosis in patients with SLE. However, with the exception of hydroxychloroquine [7] and some statins [8], the availability of beneficial treatments to decrease CVD risk in SLE is limited.

Mycophenolate mofetil (MMF) is an immunosuppressive drug used in the treatment of patients with SLE, particularly those with nephritis [9]. It is also approved to prevent transplant rejection, especially in heart and kidney transplantation. MMF is an ester pro-drug which is metabolized in the body to the active compound mycophenolic acid (MPA). MPA is a noncompetitive inhibitor of a rate-limiting purine biosynthetic enzyme, inosine-5′-monophosphate dehydrogenase (IMPDH). IMPDH is involved in *de novo* synthesis of purines, and lymphocytes rely exclusively on this *de novo* pathway for nucleotide synthesis [10], [11]. Therefore, MMF selectively targets lymphocyte proliferation. Importantly, MMF has been shown to reduce immune-mediated vascular injury in transplantation-associated atherosclerosis (known as coronary allograft vasculopathy) [12] and to attenuate plaque inflammation in patients with symptomatic carotid artery stenosis [13]. These findings further suggest a potential role for MMF in the treatment of atherosclerosis.

In the study presented here, we utilized a mouse model that displays synergy between lupus and atherosclerosis [14]. The *gld.apoE−/−* mouse model incorporates the *gld* inactivating mutation in Fas ligand (FasL) which develops lupus-like autoimmunity together with splenomegaly and lymphadenopathy; and the *apoE−/−* strain which spontaneously displays increased plasma levels of cholesterol and triglycerides and the development of atherosclerosis, particularly when mice are given a high cholesterol “Western diet”. In the study presented here, we used the *gld.apoE^−/−^* mouse model to reflect the accelerated atherosclerosis that occurs in patients with immune disorders to determine if MMF is effective in the treatment of lupus-associated atherosclerosis.

## Methods

### Animals and Study Protocol

The *gld.apoE^−/−^* mice used in this study were obtained by crossing *gld* and *apoE^−/−^* mice as previously described [14]. Starting at 7 weeks of age, the mice were maintained on Adjusted Calories Western diet (21% fat) (#88137, Harlan-Teklad) for 12 weeks, supplemented with 200 mg/kg/day mycophenolate mofetil (MMF) (n = 8) or not supplemented (control) (n = 8). The concentration was chosen based on an equivalent mouse dosage value converted from a human dosage of 2000 mg/day, using equations presented by Ng [15]. MMF was incorporated into our experimental Western diet by Harlan-Teklad Special Diets using CellCept® Oral Suspension (Roche Laboratories) and the conversion ratios we provided. This study was carried out in accordance with the recommendations in the Guide for the Care and Use of Laboratory Animals of the National Institutes of Health. The protocol was approved by the Institutional Animal Care and Use Committee of Boston University School of Medicine (Protocol number: AN-14843). All efforts were made to minimize suffering.

### Calculation of Drug Dosage in Experimental Groups

The amount of drug incorporated into the diet of each experimental group was based on established dosages of MMF used in human lupus patients. An average dose is 2000–3000 mg MMF/day, therefore for the studies presented here, we chose 2000 mg. The human dosage was converted into an equivalent dosage appropriate for mice, as metabolism of drugs differs greatly between humans and mice. Equations presented by Ng [15] were used to convert drug dosages among species of the same Class (in this case, placental mammals). First, the minimum energy cost (MEC) was established for the murine model according to the equation: 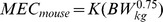; where K is a constant of 70 (for placental mammals), and BW is the body weight of the model animal (average mouse body weight is 0.025 kg). Therefore, the MEC_mouse_ value was calculated to be 4.4. Next, the MEC_mouse_ value was converted into a corresponding drug dosage. The next conversion step is to calculate a Universal MEC (UMEC) based on the MEC of a human (1694, a value established in many pharmocokinetic studies [16]) and the established human dose of 2000 mg/day. The equation to calculate this is: 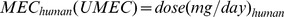; where MEC_human_ is 1694 and the drug dose is 2000 mg/day, yielding a UMEC value of 1.18. An appropriate murine dosage equivalent to the 2000 mg/day human dosage may be calculated, by using the same equation to calculate UMEC, instead using the MEC value of a mouse: 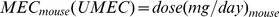; where MEC_mouse_ is 4.4 and the UMEC is 1.18, giving a murine drug dosage of 5.2 mg/day. By incorporating mouse body weight (0.025 kg) into this calculation, a value of 208 mg/kg/day is obtained. This dosage is equivalent to the 2000 mg/day human dosage (28.57 mg/kg/day, assuming a 70 kg human). According to the manufacturer’s product insert, there are 35 g of pure drug in the 110 g CellCept® oral suspension powder. Assuming that mice eat 5 g of chow per day and that the average body weight of a mouse is 0.025 kg, a ratio of CellCept® oral suspension powder that should be incorporated into each kilogram of Harlan Teklad Western diet was calculated using a pharmacokinetic equation provided by Sedgwick [16] and Gibson [12].

### Analysis of Atherosclerosis, Splenomegaly, Lymphadenopathy, and Body Weight

After 12 weeks of Western diet, the final body weights were recorded, the mice were euthanized, and the spleen and submandibular lymph nodes were removed and weighed. The heart containing the aortic root, was frozen in OCT. Frozen sections of aortic root were stained with Oil Red O solution and microphotographs were taken and values of total atherosclerotic lesion area were analyzed using Adobe Photoshop 7.0.

### Kidney Histology

Paraffin embedded tissue was sectioned (5 µm) and slides were stained with hematoxylin and eosin. Cross-sectional areas of at least 25 glomeruli were measured in each animal using computer-assisted pixel counting (Photoshop CS3; Adobe).

### Serum Measurements

Circulating anti-nuclear antibodies (ANA) were measured by immunofluorescence using HEp-2 coated slides (The Binding Site Inc., San Diego, California). Slides were incubated for 1 h with serial log-scale dilutions (1∶100 to 1∶90000; as previously described by Komori, et al.) [17] of mouse serum, washed in PBS, and then incubated with FITC-labeled goat anti-mouse IgG (whole molecule; Sigma-Aldrich, St. Louis, Missouri). Slides were viewed using fluorescent microscopy. Serum cholesterol levels were determined using a total cholesterol microtiter procedure according to the manufacturers instructions (Wako Diagnostics, Richmond VA).

### Statistical Analysis

The significant differences between mean values of atherosclerotic lesion area, spleen weight, and lymph node weight were assessed by a Mann Whitney test; a p-value <0.05 was considered significant.

## Results

### Body Weight and Food Intake

**Figure 1:**
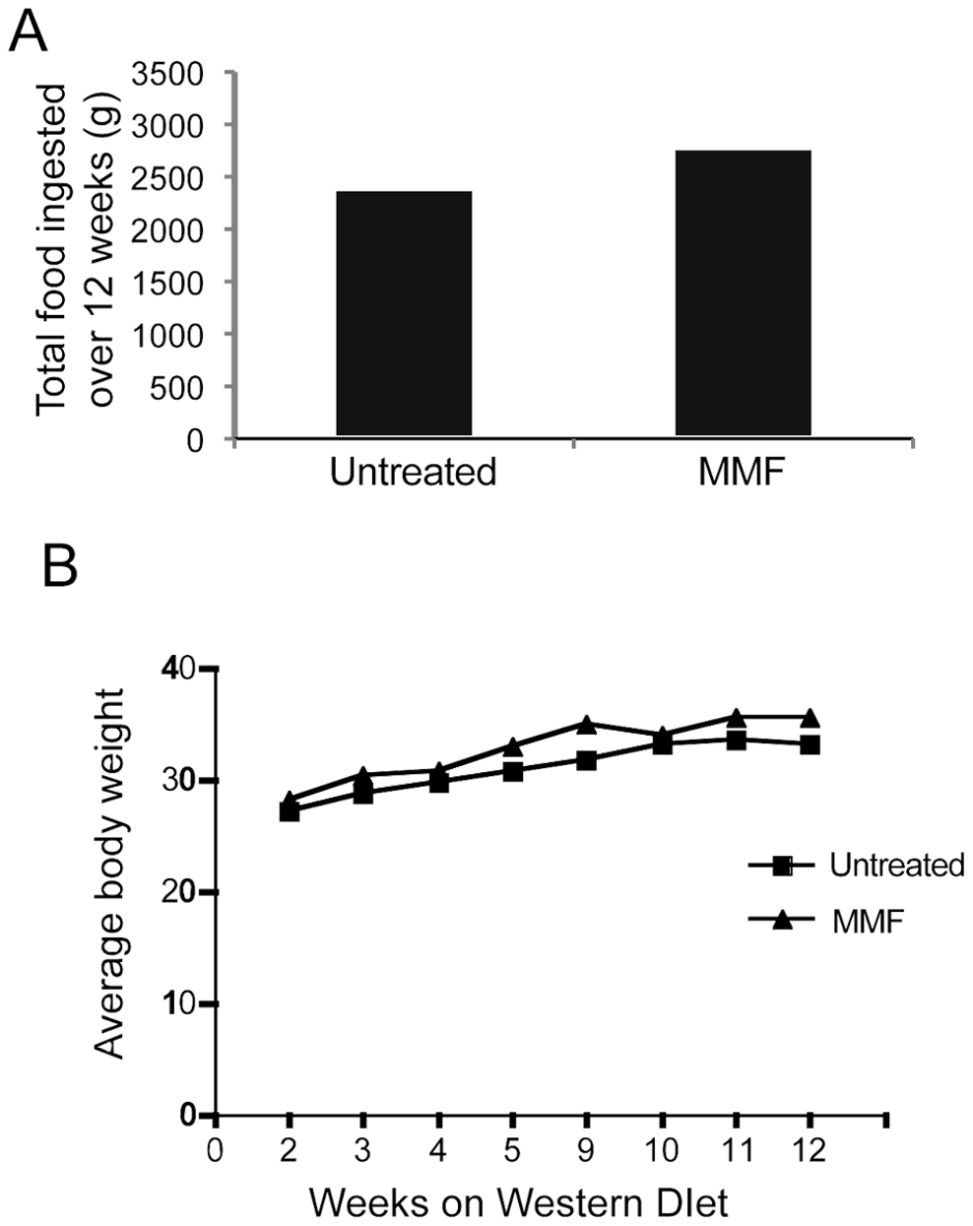
Total food intake and average body weights. *A*, Food was weighed weekly and total amount ingested was recorded for each group (control, n = 8; MMF, n = 9). *B,* Evolution of body weight as weighed weekly during the course of the experiment.

MMF has an excellent oral bioavailability. However, this treatment frequently induces gastrointestinal symptoms in humans [18], which could decrease drug intake. In order to ensure that the mice in each experimental group were ingesting similar amounts of food, the amount of food ingested was recorded weekly over the course of the experiment, and total ingestion of each group is presented in Figure 1A. Total body weights of the mice were also measured at intervals throughout the experiment (Figure 1B). There was no difference between the mice treated with MMF and control mice with regard to total food intake or body weight.

### The Development of Splenomegaly, Lymphadenopathy and Autoantibodies are Inhibited by MMF Treatment

**Figure 2:**
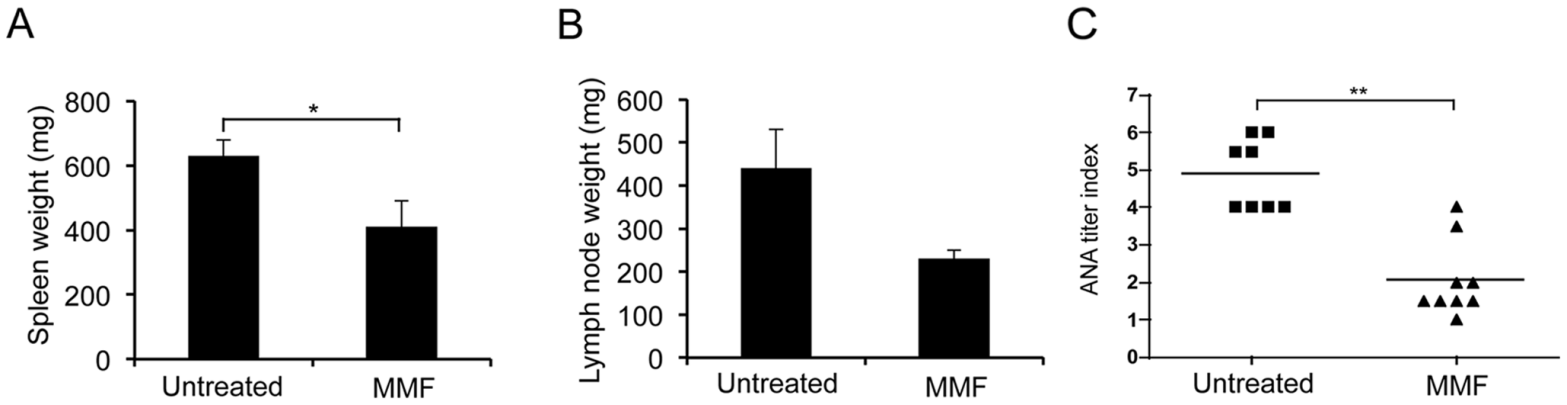
MMF treatment decreases the autoimmune phenotype in *gld.apoE−/−* mice. *A,* Spleen from mice, treated with 200 mg/kg/day MMF or untreated, was harvested and weighed (*, *p*<0.05). *B,* The lymph nodes were also harvested and weighed. *C,* ANA titer was determined from serum samples using HEp-2-coated slides and scored using the value of the last positive dilution (**, *p = *0.001). Data are expressed as means ± SEM.

After 12 weeks treatment, the spleen and submandibular lymph nodes of the *gld.apoE^−/−^* mice were removed and weighed. The mice receiving MMF had a significantly smaller spleen weight compared to mice not receiving MMF (control): 0.41±0.08 g versus 0.63±0.05 g in the MMF group (p = 0.02, using Mann Whitney test) (Figure 2A), as well as a lower, but not significant, lymph node weight compared to control (mean ± SEM): 0.23±0.02 g versus 0.44±0.09 g, respectively (p = 0.09) (Figure 2B). To determine if MMF effects were due to a modification of lymphocyte population, we performed immunohistochemical staining for T-cells and B-cells in lymph node sections from treated and untreated mice. Similar to a previous report [19], no significant effect was observed in the appearance or distribution of T- or B-cells within the lymph nodes regardless of treatment (data not shown). We then examined the effect of MMF dose on autoantibody production. In humans, a decrease in autoantibody titers has been shown during the course of MMF therapy [9]. We found a similar result in our lupus-associated atherosclerosis mouse model with a significant decrease of anti-nuclear antibody (ANA) titer in the MMF group compared to the controls (median [IQR]): 1.5 [1.5–2] versus 4 [4–5.5], respectively (p = 0,001) (Figure 2C).

### Improvement of Nephritis in gld.apoE^−/−^ Mice Receiving MMF

**Figure 3:**
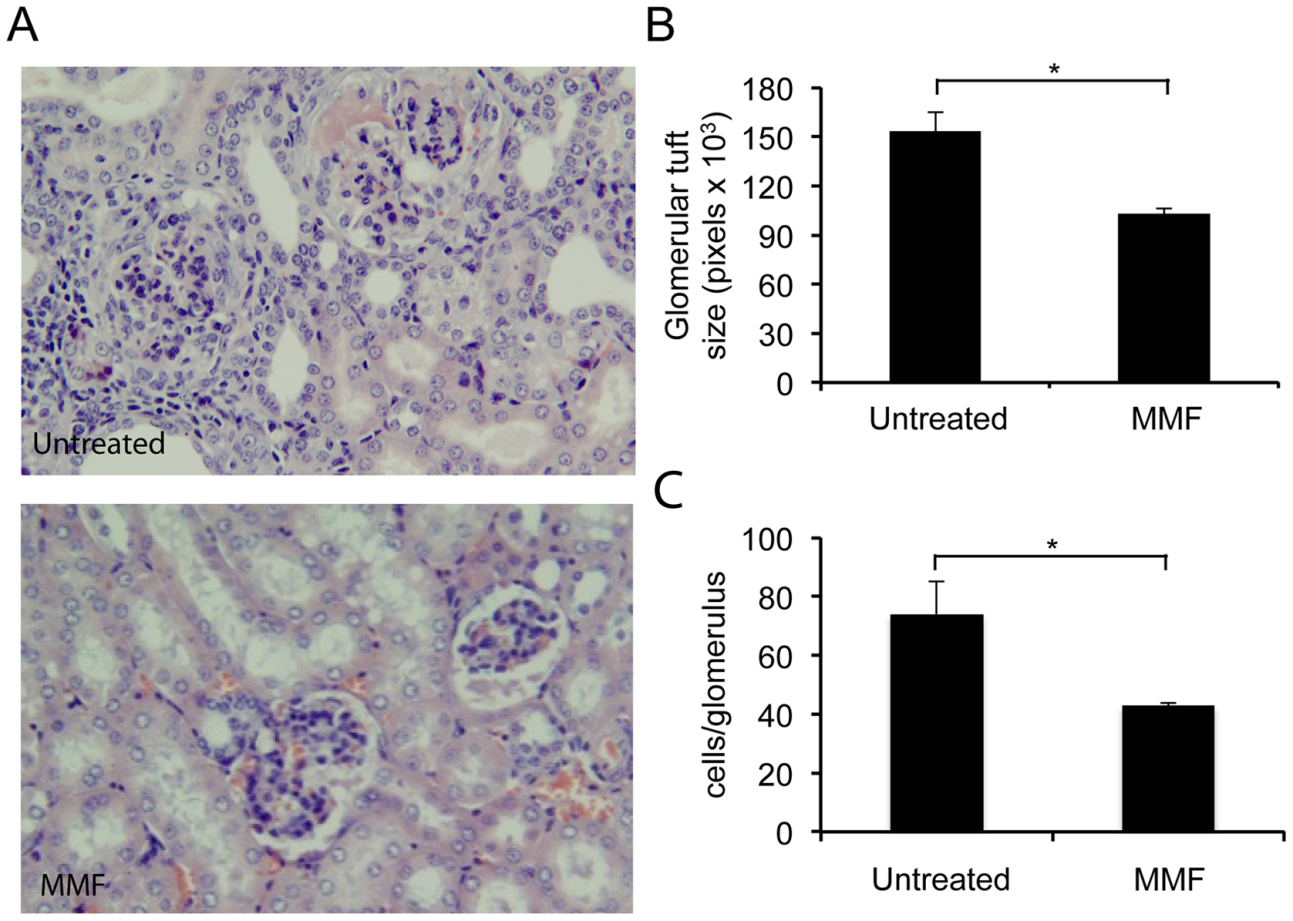
Renal disease is ameliorated in *gld.apoE−/−* mice after MMF treatment. The mice were treated with MMF or control for 12 wk. *A,* Representative H&E-stained sections of kidney in both groups. Glomerular tuft size (*B*) (*, *p = *0.002) and cell count (*C*) (**, *p = *0.004) were measured by computer-assisted pixel counting. Values shown are the mean ± SEM.

MMF is currently used as induction therapy, maintenance therapy, or both for patients with lupus nephritis [9], [20], [21], [22], [23]. Although B6 mice deficient in Fas (*lpr*) or FasL (*gld*) display mild or no kidney disease [24], the lack of apoE associated with the *gld* mutation induces significant kidney disease [14]. We assessed the extent of renal damage associated with the lupus phenotype, in control versus the MMF treated experimental *gld.apoE^−/−^* group. Glomerular tuft size and cell count were significantly decreased in *gld.apoE^−/−^* after treatment with MMF (respectively, p = 0.002 and p = 0.004) (Figure 3A–C). Of note, infiltration of inflammatory cells as well as modest crescent formation was observed in control mice, but not in MMF treated mice. Our findings demonstrate the efficacy of MMF on lupus parameters in our mouse model, as previously described in human SLE and other mouse models.

### MMF Decreases the Severity of Atherosclerosis in gld.apoE^−/−^ Mice

**Figure 4:**
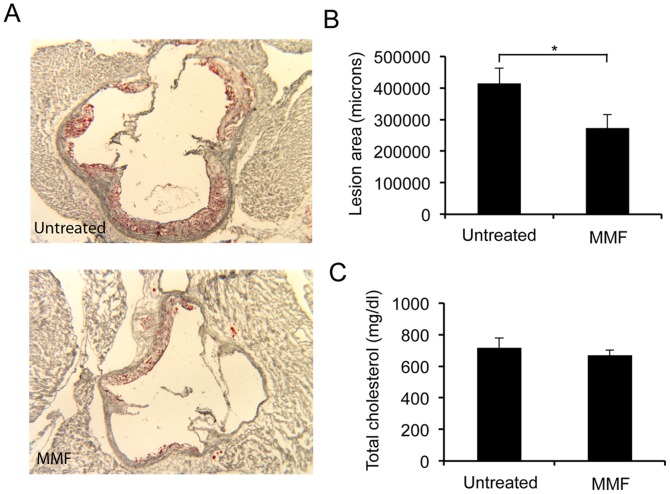
Decreased atherosclerosis in *gld.apoE−/−* mice treated with MMF. Aortic root atherosclerotic lesion area in MMF-treated or control mice. *A,* Representative photographs of aortic root stained with Oil Red O from mice maintained on Western diet for 12 wk and treated with 200 mg/kg/day MMF or untreated. *B,* Atherosclerotic lesion area of Oil Red O-stained aortae was quantified in both groups (*, *p = *0.02). *C*, Total serum cholesterol was quantified in control mice and mice treated with 200 mg/kg/day MMF.

To determine the effect of MMF on atherosclerotic parameters, the aortic root of each mouse was stained with Oil red O to reveal areas of atherosclerotic lesion after 12 weeks on Western diet (WD), (Figure 4A). A significant decrease in lesion area was observed in the MMF-treated mice compared to control (Figure 4B) (p = 0.02). Serum levels of total cholesterol were also examined to determine whether the protective effects of MMF on atherosclerosis severity could be explained by differences in serum cholesterol levels. However, there was no detectable difference between mice treated with MMF compared to control: 716±63 mg/dl versus 669±32 mg/dl, respectively (p = 0.38) (Figure 4C).

## Discussion

The purpose of this experiment was to investigate the effects of mycophenolate mofetil (MMF), on development of premature atherosclerosis in a murine mouse model of accelerated atherosclerosis and systemic lupus erythematosus (SLE). The *gld.apoE^−/−^* model is ideal to use for this experiment based on previous findings of synergistic disease presentation of both SLE and atherosclerosis in mice [14]. Our study investigated the effect of a physiologically relevant dose of MMF on disease development. The 200 mg/kg/day concentration of MMF in mouse diet is roughly equivalent to the 2000 mg/day dose of MMF approved for use in human renal transplant prophylaxis and commonly used for the treatment of human SLE. Severity of cardiovascular disease was assessed by quantifying the atherosclerotic lesion area in the aortic root. To assess the hallmarks of SLE associated with the *gld.apoE^−/−^* model, spleen and submandibular lymph node were weighed. As such, the data show that the dosage of 200 mg/kg/day yields a significant ameliorating effect on atherosclerosis, splenomegaly and lymphadenopathy presentation. These data suggest that MMF treatment of patients with SLE could not only be beneficial to lupus, but also decrease the risk of cardiovascular disease.

With advances in medical care, the quality of life has improved and survival rate has increased for patients with SLE [2]. However, with this increased survival rate, there is also a correlated increase in CVD. Chronic inflammation has been implicated as a contributing factor for the development of premature atherosclerosis in SLE patients [3], [25], [26], [27]. Therefore, there is an interest in using anti-inflammatory or immunomodulatory therapies for this condition. MMF, an immunosuppressive agent, is currently used for the treatment of SLE patients, particularly those with kidney involvement, as well as for the prevention of rejection in transplant patients.

MMF was first demonstrated to inhibit T-cell function, however, MMF also exerts inhibitory effects on other immune cells and effectors, including downregulation of cell adhesion molecules and attenuation of monocyte and macrophage responses [28]. MMF has also been shown to suppress a number of the inflammatory events that are involved in the development of atherosclerosis. T-lymphocyte infiltration to atherosclerotic plaque and circulation to sites of inflammation is abrogated by MMF treatment [28]. MMF has been shown to reduce the expression of vascular adhesion molecules in atherosclerosis by inhibiting the nuclear factor NFκB which is required for their transcriptional upregulation [29]. Raisanen *et al* [30] showed that MMF treatment reduces the appearance and proliferation of smooth muscle cells in the intima, which normally contribute to atherosclerotic plaque formation by recruitment of extracellular matrix and self-proliferation. Thus, MMF has properties that could be considered anti-atherogenic: inhibiting T-cells, blocking leukocyte adhesion and inhibiting proliferation of smooth muscle cells; therefore making it a potentially valuable drug to prevent the development of atherosclerosis in patients with SLE.

A recent publication examined the effect of MMF on atherosclerosis development by using bone marrow transplantation to generate a mouse model of lupus with associated atherosclerosis [31]. van Leuven et al. report that the LDLr*−/−*. Sle mouse shows decreased atherosclerosis after MMF treatment, although lesion area does not decrease with atorvastatin treatment alone. However, despite the beneficial effects to atherosclerosis, there are no discernable differences in circulating autoantibody levels or kidney pathology. While it is known that B6 mice deficient in Fas (*lpr*) or FasL (*gld*) display mild or no kidney disease, the *gld.apoE^−/−^* model develops glomerular tuft enlargement and significant inflammatory cell infiltrate to the kidney [14]. The results presented in the current study show a decrease in both lupus-disease and cardiovascular disease. It is reasonable to suggest that differences in the disease models utilized (*Ldlr−/−* versus *apoE−/−*, respectively*;* and *Sle1.2.3* versus *gld*, respectively) or the dosage (40 mg/kg/day versus 200 mg/kg/day) could have contributed to the contrasting results.

Substantial evidence exists to support a critical role of inflammation in the pathology of both SLE and CVD caused by atherosclerosis. The chronic inflammation associated with SLE correlates to the increased risk in CVD seen in patients [25], [26], [27]. The current study suggests that MMF, in addition to this well-known efficacy on lupus nephritis, might be a promising agent for the prevention of atherosclerosis in SLE. A more extensive analysis of atherosclerosis and SLE in these MMF-treated *gld.apoE^−/−^* mice will need to be performed to fully determine how MMF impacts the accelerated atherosclerosis of our lupus mouse model and potentially bring a better understanding of the link between atherogenesis and autoimmune disease.
